# ﻿Marsupials (Didelphidae, Mammalia) of Mato Grosso do Sul state (Brazil): taxonomic accounts, species richness, and biogeography

**DOI:** 10.3897/zookeys.1243.141601

**Published:** 2025-06-23

**Authors:** Nilton C. Cáceres, Geruza L. Melo, Jonas Sponchiado, Gabriel M. Martin

**Affiliations:** 1 Departamento de Ecologia e Evolução, CCNE, Universidade Federal de Santa Maria, Santa Maria, RS, Brazil Universidade Federal de Santa Maria Santa Maria Brazil; 2 Instituto Federal de Educação, Ciência e Tecnologia Farroupilha – IFFAR – Campus Alegrete, Alegrete, RS, Brazil Instituto Federal de Educação, Ciência e Tecnologia Farroupilha Alegrete Brazil; 3 Centro de Investigación Esquel de Montaña y Estepa Patagónica (CIEMEP), Consejo Nacional de Investigaciones Científicas y Técnicas (CONICET) y Universidad Nacional de la Patagonia “San Juan Bosco” (UNPSJB), Esquel, Chubut, Argentina Universidad Nacional de la Patagonia “San Juan Bosco” Esquel Argentina

**Keywords:** Atlantic forest, Cerrado woodland, Chiquitano forest, museum specimens, opossums, Pantanal wetland, species turnover, temperature gradient

## Abstract

The marsupials of Mato Grosso do Sul (MS) state in southwestern Brazil are still poorly known, with most research being concentrated around the Pantanal wetland. In this work, the marsupial richness was analysed in four different ecoregions of MS, based on more than ten years of sampling using live and pitfall traps, comparing them with published information. Fifteen marsupial species were recorded, adding 117 new records and increasing the previously known richness by more than 50%. These new records represent an increase between 96.7% (*Gracilinanusagilis*) to 9.1% (*Chironectesminimus*) of those previously known for the state, with an average increase of 43%. *Cryptonanusagricolai* is recorded for the first time for MS, but we did not trap *Caluromyslanatus* and *Metachirusmyosuros*, which were mentioned in the literature. The Cerrado ecoregion (a type of savanna) shows more species than other ecoregions, being the largest ecoregion in the state. A strong faunal turnover was found in the state, from the humid and mild forests of the southeast (Atlantic forest ecoregion) to the more seasonal, dry, and warm forests, shrublands, and grasslands of the northwest (Chiquitano and Pantanal ecoregions). A full taxonomic account and localities of the species recorded are provided and the biogeographical affinities of ecoregions present in MS are discussed.

## ﻿Introduction

The patterns of species diversity of a given region are influenced by several factors including altitude (McCain 2004), vegetation type (Cáceres et al. 2014), climate (Dambros et al. 2015), and historical patterns (Vivo and Carmignotto 2004). However, one of the primary causes to determine a higher local faunal diversity could be vegetation complexity (Pardini et al. 2005).

Being mostly tropical, Brazil is considered a megadiverse country (Quintela et al. 2020). Its high biological diversity is in part attributed to the presence of several phytogeographic domains, including Amazon forest, Atlantic forest, and Cerrado, among others (Fiaschi and Pirani 2009). This scenario leads to a high diversity of mammal species in the country (Quintela et al. 2020). Among them, Brazil has 65 species of marsupials, being the richest country in America considering this group (Astúa et al. 2023). All of them belong to the family Didelphidae and their distribution shows a major species richness in the Atlantic forest, in the easternmost portion of the country (Martin et al. 2023).

The southwestern region of Brazil, specifically Mato Grosso do Sul (MS) state, harbours a significant portion of Brazil’s marsupial diversity, reflecting the region’s high heterogeneity in vegetation types (Cáceres et al. 2008). The Cerrado is the main ecoregion of MS (while “cerradão” is the most common vegetation in the region), which also includes the Pantanal wetland in the west and the Atlantic forest in the southeast. To a lesser extent but still important is the Chiquitano Forest in the northwestern corner of the state, on the border with Bolivia (Veloso et al. 1991). Importantly, the western portion of the state (west of the Maracaju plateau in the Paraguay River basin) has been much more sampled in terms of mammalian and marsupial diversity than other regions, particularly because of the overall research focus on the Pantanal wetlands and their biological diversity (e.g., Lacher and Alho 1989; Trolle 2003; Cáceres et al. 2007a, 2007b, 2008, 2010; Andreazzi et al. 2011; Tomás et al. 2011; Hannibal et al. 2017; Antunes et al. 2021).

**Figure 1.**
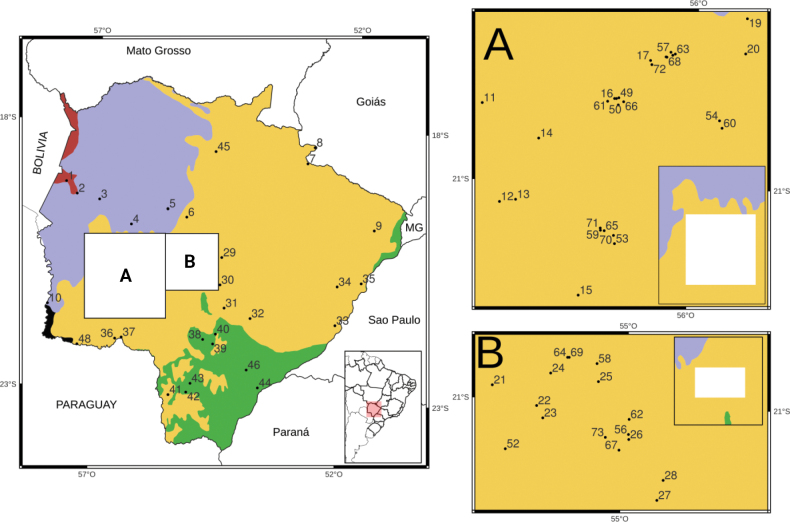
Map showing the study area (Mato Grosso do Sul state) and localities sampled, as numbered in Table 1. Ecoregions (sensu Dinerstein et al. 2017) are shown using different colours: Chiquitano forest (red), Pantanal wetlands (purple), Chaco (black), Cerrado (yellow/orange), Atlantic forest (green). See Table 1 for locality names, geographic coordinates, and species number at the museum collection (“Coleção de Mamíferos da Universidade Federal de Santa Maria”, Brazil).

Biogeographically, transitional regions can be characterised by high species richness due to the overlap of species distributional ranges, being composed of inhabitants of adjacent biomes and presenting a high level of endemism provided by the uniqueness of regions (Silva 1995; Ratter et al. 2003). In addition, rivers and mountains can function as geographical barriers, being important factors in limiting species distributions (Sánchez-Cordero 2001; McCain 2004). Mato Grosso do Sul state is located between two large rivers (Paraná and Paraguay), with its western portion being mostly plain (including most of the Pantanal wetlands, with altitudes around 100 m a.s.l.), but presenting different plateaus and “sierras”, such as Serra da Bodoquena, Serra de Maracaju, and Serra do Urucum, with altitudes varying from 500 to 1,000 m a.s.l. (Fig. 1).

Given the diversity of marsupials in Brazil (Astúa et al. 2023) and several biogeographic and environmental factors that can influence their distribution, it is important to evaluate how these factors influence their species composition and richness, especially in complex regions of the country where there are different ecoregions, mountains, and river basins. Our objective is to analyse the marsupial species richness, taxonomic composition, and biogeographic affinities in the different ecoregions present in MS state. We predict that there will be a strong faunistic gradient of marsupial composition, following the ecoregional heterogeneity and regional climate, with richness increasing towards the southeast (Atlantic forest), far from the Pantanal wetlands.

## ﻿Materials and methods

For the purpose of this study, we follow the ecoregional division of MS state as proposed by Dinerstein et al. (2017) (Fig. 1). The state can be divided according to two hydrographic basins (Paraná and Paraguay basins), with the Paraguay basin in the west being lower in altitude, warmer, and drier (Marcuzzo and Costa 2012; Aparecido et al. 2020; Ivasko et al. 2020). Most of the Pantanal wetland occurs within the state, westward (in the Paraguay River basin), which is usually called “Southern Pantanal” (Trolle 2003); it is periodically flooded during the summer months. The central region of the state is composed of woodland savanna (named here as Cerrado ecoregion), which can be subdivided in western Cerrado (Paraguay basin) and eastern Cerrado (Paraná basin). A reasonable portion of seasonal, deciduous Atlantic forest is present in the southeast of the state; this ecoregion (named here as Atlantic forest) does regularly form mosaics with the Cerrado, in a transitional zone (Ivasko et al. 2020). Finally, the extreme northwest region of the state includes the Chiquitano forest ecoregion, at the western margin of the Paraguay River, connected to deciduous forests of eastern Bolivia (Fig. 1). All the state has a deficit of rains to some extent, mainly in the west and north, between the months of April to September (Marcuzzo and Costa 2012; Aparecido et al. 2020; Ivasko et al. 2020). A very small portion of the Chaco ecoregion is present in the extreme southwestern portion of the state, which we did not sample.

Marsupial species were recorded by locality and ecoregion in MS state. We sampled small mammals mostly throughout 12 years (2001 to 2013), using live-traps and pit-fall traps with a preference for the former (89% localities exclusively sampled with live traps – Sherman or Tomahawk), although pit-fall traps were used in 11% of all areas (exclusively or combined with live traps; Table 1). In each sampled area (a woodland fragment or riparian forest), we usually used 2–5 transect lines (average of 3) with 10–15 live traps in each one (averaging 12.75 traps), usually spaced 10 m apart from each other. Although used less frequently, pit-fall traps had buckets with 30–100 litres, usually spaced 10 m from each other and interconnected by an 80–100 cm height drift-fence. Overall, areas were sampled for four consecutive nights (51%) or 20 non-consecutive nights (47%), averaging 12.5 trapping nights; this difference in sampling was accounted for when we used quadrats (with ~ 55 km in length) encompassing different areas for analyses (see below). Voucher specimens for each area were mostly deposited in the Mammal Collection at Universidade Federal de Santa Maria (UFSM) (Table 1), from where we analysed all marsupial species considered in this manuscript (plus those from the literature). All procedures performed were part of the routine of animal care, following the American Guidelines for Animal Care and Use (Sikes et al. 2011).

**Table 1.** List of most localities (51 of 73) sampled in Mato Grosso do Sul state, Brazil, with their geographical coordinates and local species richness. Localities with voucher specimens are preferentially shown. Species sampled are shown per locality (v = voucher; r = released in the field; ph = with photo). UFSM museum (Mammal Collection at Universidade Federal de Santa Maria) voucher number and sex (M or F) are provided; when the specimen was deposited in another museum, we indicate it by using the respective acronym [UFMT (Universidade Federal do Mato Grosso at Cuiabá); MHNCI (Museu de História Natural Capão da Imbuia at Curitiba)]. Number of days sampled within each locality is shown in brackets.

Locality name and county	Coordinates	Altitude (m a.s.l.) / Ecoregion	Sampled species	Museum voucher	Sampling effort /day
1. Morro Santa Cruz – Corumbá	19°10'13"S, 57°36'59"W	505 m/ Chiquitano forest	*C.chacoensis* (v)	267 (F)	9 transect lines (8 pitfall traps per line) [60]
			*M.ocellatus* (v) (ph)	605 (M)	
			*M.kunsi* (v)	696 (M)	
			*M.rapposa* (v)	789 (M)	
			*M.domestica* (v)	717 (F)	
2. Albuquerque – Corumbá	19°23'50"S, 57°23'55"W	166 m/ Chiquitano forest	*M.domestica* (v)	559 (M)	5 transect lines (13 live traps per line) [4]
			*G.agilis* (r)	–	
			*D.poecilotis* (r)	–	
3. Fazenda Xaraés – Corumbá	19°29'32"S, 56°57'13"W	94 m/ Pantanal	*G.agilis* (v) (ph)	557 (F)	5 transect lines (13 live traps per line) [4]
			*P.canus* (r) (ph)	–	
			*D.poecilotis* (r) (ph)	–	
4. Fazenda Caiman – Miranda	19°56'14"S, 56°18'35"W	127 m/ Pantanal	*G.agilis* (r)	– (ph)	5 transect lines (13 live traps per line) [4]
			*P.canus* (r)	– (ph)	
5. Fazenda Santana – Aquidauana	19°37'20"S, 55°36'16"W	123 m/ Pantanal	*M.domestica* (v)	373 (M)	2 transect lines (30 live traps per line)
			*M.murina* (v)	372 (–)	1 transect lines (6 pitfall traps per line) [4]
			*G.agilis* (r)	–	
6. Fazenda Rodeio – Corguinho	19°45'57"S, 55°13'34"W	334 m/ Cerrado	*G.agilis* (v)	371 (F)	3 transect lines (30 live traps per line)
					2 transect lines (6 pitfall traps per line) [4]
7. Fazenda Pouso Frio – Chapadão do Sul	18°39'46"S, 52°54'57"W	834 m/ Cerrado	*G.agilis* (v)	252 (F)	5 transect lines (20 live traps per line) [4]
			*D.poecilotis* (r)	–	
8. Fazenda Santo Antônio - Costa Rica	18°21'23"S, 52°47'38"W	807 m/ Cerrado	*M.murina* (v)	325 (–)	5 transect lines (20 live traps per line) [4]
			*D.poecilotis* (r)	–	
9. Fazenda Lindos Campos – Inocência	19°49'24"S, 51°32'49"W	426 m/ Cerrado	*L.crassicaudata* (v)	326 (F)	5 transect lines (12 live traps per line) [4]
			*T.macrurus* (v)	487 (F)	
10. Porto Conceição - Porto Murtinho	21°27'55"S, 57°55'04"W	86 m/ Pantanal	*G.agilis* (r) (ph)	–	5 transect lines (13 live traps per line) [4]
			*T.macrurus* (r) (ph)	–	
			*M.domestica* (r) (ph)	–	
11. Fazenda Califórnia – Bodoquena	20°42'19"S, 56°52'13"W	470 m/ Cerrado	*M.domestica* (v)	029 (M)	2 transect lines (15 live traps per line) [6]
12. Fazenda Princesinha – Bonito	21°05'04"S, 56°47'02"W	558 m/ Cerrado	*M.domestica* (v) (ph)	010 (F)	3 transect lines (15 live traps per line) [5]
			*M.rapposa* (v)	006 (F)	
			*T.macrurus* (v)	005 (M)	
13. Fazenda Santa Tereza – Bonito	21°04'25"S, 56°43'05"W	497 m/ Cerrado	*G.agilis* (r)	–	5 transect lines (13 live traps per line) [4]
14. Fazenda Santa Terezinha – Bonito	20°50'02"S, 56°37'59"W	615 m/ Cerrado	*T.macrurus* (v)	035 (M)	1 transect line (30 live traps per line) [3]
15. Recanto Rio da Prata – Jardim	21°26'01"S, 56°26'40"W	281 m/ Cerrado	*G.agilis* (r)	–	5 transect lines (13 live traps per line) [4]
16. Fazenda Santa Maria – Bonito	20°40'10"S, 56°19'43"W	184 m/ Cerrado	*M.rapposa* (r)	–	2 transect lines (10 live traps per line) [20]
17. Fazenda Borboleta – Miranda	20°31'00"S, 56°11'16"W	194 m/ Cerrado	*G.agilis* (r) (ph)	–	5 transect lines (13 live traps per line) [4]
			*T.macrurus* (r) (ph)	–	
18. Fazenda Campo Alegre – Anastácio	20°29'35"S, 56°05'52"W	223 m/ Cerrado	*M.rapposa* (v) (ph) *T.macrurus* (v)	628 (M) UFMT	2 transect lines (10 live traps per line) [20]
19. UEMS – Aquidauana	20°20'24"S, 55°47'58"W	214 m/ Cerrado	*G.agilis* (v)	220 (M)	1 transect line (15 live traps per line) [4]
			*M.domestica* (v)	040 (F)	
20. Centre square – Aquidauana	20°28'33"S, 55°48'00"W	149 m/ Cerrado	*C.philander* (v)	234 (F)	accidental capture
21. Piraputanga – Aquidauana	20°27'16"S, 55°29'53"W	198 m/ Cerrado	*D.poecilotis* (v)	046 (–)	1 transect line (20 live traps per line) [20]
			*M.murina* (v)	536 (F)	
			*T.macrurus* (v)	049 (F)	
22. Fazenda Santa Helena - Dois Irmãos do Buriti	-20.5201, -55.329171	315 m/ Cerrado	*C.chacoensis* (v)	477 (F)	02 trap grids (100 live traps per grid)
			*T.macrurus* (v)	678 (M)	08 transect lines (10 pitfall traps per line) [20]
23. Fazenda São Cristóvão - Dois Irmãos do Buriti	-20.562517, -55.304065	325 m/ Cerrado	*G.agilis* (v) (ph)	207 (M)	05 trap grids (100 live traps per grid)
			*M.domestica* (r)	–	08 transect lines (10 pitfall traps per line) [20]
			*M.rapposa* (v)	534 (M)	
			*M.kunsi* (v)	167 (M)	
			*T.macrurus* (v)	359 (M)	
24. Fazenda Cachoeirão – Terenos	20°24'13"S, 55°16'57"W	302 m/ Cerrado	*C.chacoensis* (v)	647 (M)	02 trap grids (100 live traps per grid)
					08 transect lines (10 pitfall traps per line) [20]
25. Fazenda Primavera – Terenos	20°25'33"S, 55°06'11"W	308 m/ Cerrado	*T.macrurus* (v)	631 (F)	2 transect lines (10 live traps per line) [20]
26. Fazenda Sucuri – Terenos	20°37'26"S, 54°58'46"W	267 m/ Cerrado	*M.murina* (v)	634 (F)	2 transect lines (10 live traps per line) [20]
27. Fazenda Serrinha – Sidrolândia	20°49'58"S, 54°51'48"W	444 m/ Cerrado	*C.minimus* (v)	031 (F)	accidental capture
28. Fazenda Nova Esperança – Sidrolândia	20°45'41"S, 54°50'37"W	507 m/ Cerrado	*D.poecilotis* (r)	–	5 transect lines (13 live traps per line) [4]
			*G.agilis* (r)	–	
29. Fazenda Sossego - Campo Grande	20°29'05"S, 54°29'58"W	619 m/ Cerrado	*G.agilis* (v) (ph)	555 (M)	5 transect lines (13 live traps per line) [4]
			*T.macrurus* (v) (ph)	554 (F)	
30. Anhanduí - Campo Grande	20°59'40"S, 54°30'23"W	420 m/ Cerrado	*D.poecilotis* (r)	–	5 transect lines (12 live traps per line) [4]
			*D.aurita* (r)	–	
31. Fazenda Bela Vista - Nova Alvorada do Sul	21°25'15"S, 54°24'02"W	455 m/ Cerrado	*D.poecilotis* (v)	245 (M)	5 transect lines (20 live traps per line)
					1 transect line (4 pitfall traps per line) [4]
32. Fazenda Laranjeira - Nova Alvorada do Sul	21°35'16"S, 53°52'28"W	350 m/ Cerrado	*D.poecilotis* (r)	–	5 transect lines (13 live traps per line) [4]
			*G.agilis* (r)	–	
33. Fazenda Conquista - Santa Rita do Pardo	21°37'08"S, 52°11'19"W	305 m/ Atlantic forest	*D.poecilotis* (r)	045 (F)	5 transect lines (20 live traps per line)
					1 transect line (5 pitfall traps per line) [4]
34. Distrito de Rio Verde - Três Lagoas	20°54'03"S, 52°11'50"W	361 m/ Cerrado	*D.poecilotis* (r)	–	5 transect lines (13 live traps per line) [4]
			*G.agilis* (r)	–	
35. Estância Figueira - Três Lagoas	20°48'50"S, 51°43'50"W	325 m/ Atlantic forest	*C.agricolai* (v)	089 (M)	5 transect lines (18 live traps per line)
			*G.agilis* (v)	085 (M)	1 transect line (7 pitfall traps per line) [4]
			*D.poecilotis* (r)	–	
36. Granja, Exército Brasileiro - Bela Vista	22°04'45"S, 56°33'00"W	238 m/ Cerrado	*D.poecilotis* (r) (ph)	–	5 transect lines (13 live traps per line) [4]
			*G.agilis* (r)	–	
			*M.rapposa* (r) (ph)	–	
			*T.macrurus* (r) (ph)	–	
37. Fazenda Redomão - Bela Vista	22°03'10"S, 56°25'15"W	276 m/ Cerrado	*M.rapposa* (r)	–	5 transect lines (10 live traps per line) [4]
38. Fazenda Lagoão – Itaporã	22°01'29"S, 54°47'31"W	354 m/ Atlantic forest	*C.chacoensis* (v)	MHNCI	2 transect lines (5 live traps per line) [4]
39. Fazenda Monjolo – Douradina	22°05'56"S, 54°35'20"W	309 m/ Atlantic forest	*G.agilis* (v) (ph)	553 (F)	2 transect lines (5 live traps per line) [4]
			*D.poecilotis* (r) (ph)	–	
40. Fazenda Inho - Rio Brilhante	21°54'49"S, 54°32'40"W	285 m/ Atlantic forest	*C.agricolai* (v)	019 (M)	2 transect lines (5 live traps per line) [4]
			*D.poecilotis* (r)	–	
41. Escola Agrícola – Amambai	23°05'06"S, 55°25'38"W	445 m/ Atlantic forest	*G.agilis* (v)	469 (F)	1 transect line (5 pitfall traps) [2]
42. Fazenda Alegrete – Amambai	23°01'12"S, 55°04'21"W	383 m/ Atlantic forest	*D.poecilotis* (r)	–	5 transect lines (13 live traps per line) [4]
			*G.agilis* (r)	–	
43. Fazenda Campanário - Laguna Carapã	22°51'08"S, 54°59'49"W	364 m/ Atlantic forest	*D.poecilotis* (r) (ph)	–	5 transect lines (13 live traps per line) [4]
44. Parque Estadual das Várzeas do Rio Ivinhema – Naviraí	22°51'52"S, 53°38'38"W	303 m/ Atlantic forest	*G.agilis* (v)	613 (M)	5 transect lines (13 live traps per line) [4]
			*M.murina* (v) (ph)	612 (M)	
45. 47o. Batalhão do Exército – Coxim	18°31'12"S, 54°42'00"W	245 m/ Cerrado	*G.agilis* (v)	912 (–)	2 transect lines (12 live traps per line) [24]
46. Fazenda Japema - Novo Horizonte do Sul	22°32'44"S, 53°53'23"W	387 m/ Atlantic forest	*M.murina* (v)	903 (M)	2 transect lines (15 live traps per line) [4]
47. Pousada das Amoras (São Lourenço) - Aquidauana	19°37'44"S, 55°36'12"W	130 m/ Pantanal	*G.agilis* (v)	509 (F)	1 transect lines (30 live traps per line)
					1 transect lines (6 pitfall traps per line) [4]
48. Base do Exército de Bela Vista – Caracol	22°12'49"S, 57°18'03"W	127 m/ Cerrado	*P.canus* (r) (ph)	–	2 transect lines (15 live traps per line) [4]
49. Águas de Miranda - Bonito	20°39'58"S, 56°18'40"W	Cerrado	*C.chacoensis* (v)	UFMT	2 transect lines (10 live traps per line) [20]
			*M.domestica* (r)	–	
50. Águas de Miranda - Bonito	20°41'38"S, 56°18'44"W	Cerrado	*G.agilis* (v)	UFMT	2 transect lines (10 live traps per line) [20]
			*M.rapposa* (v)	625 (M)	
			*T.macrurus* (r)	–	
51. Fazenda Campo Alegre - Miranda	20°30'02"S, 56°07'30"W	Cerrado	*G.agilis* (r)	–	2 transect lines (10 live traps per line) [20]
			*M.domestica* (r)	–	
			*T.macrurus* (v)	636 (M)	

Species richness and composition were calculated in grids of 0.5° × 0.5° quadrats using DIVA-GIS 7.5.0 (Hijmans et al. 2005) point-to-grid analysis, and records were used to calculate ecoregional richness with the point-to-polygon analyses. Other spatial analyses and most figures were generated using QGIS v. 3.30 (QGIS.org 2023).

For a comparative approach, we revised the literature for reports of marsupial species in MS state (such database is available with the authors under reasonable request) and used this for an integrative analysis by comparing our field data with those from the literature, thereby measuring our sampling effectiveness (e.g., our percent record increase in each ecoregion compared to the literature).

Taxonomic, biogeographic and ecological notes are provided for each species, when relevant. Additional information on taxonomy and primary synonymy of each species can be found in Astúa et al. (2023) and Chemisquy et al. (2025) for *Didelphispoecilotis*.

We made biogeographic analyses using ecoregions to categorise grid quadrats, throughout MS state. These biogeographic analyses were made using Principal Coordinate Analysis (PCoA) to relate our species composition per quadrat (0.5° × 0.5°) with every other quadrat, investigating species composition affinities across ecoregions and climates. Climatic variables (mean annual temperature and annual precipitation) were obtained for each quadrat centroid from Bioclim (http://www.worldclim.org/bioclim). We used presence-absence data for species within quadrats and the Jaccard index of similarity for PCoA. Only species present in at least two quadrats and quadrats with at least three species were considered. In addition, we performed Pearson correlation analyses between each PCoA axis and climate in order to examine what climatic variable (mean annual temperature and annual precipitation) better correlated with the marsupial community turnover (across different quadrats and captured by PCoA axes) across the state. The best climatic variable was shown in the PCoA plot. Finally, we ran a CCA analysis (Correspondence Canonical Analysis) to search for the climatic correlation (mean annual temperature and annual precipitation) with the species turnover across the sampled quadrats; the dataset was the same as for the PCoA analysis but including climatic data. All statistical analyses were made using PAST software v. 4.01 (Hammer et al. 2001).

## ﻿Results

### ﻿Species sampled, localities, and ecoregions

We recorded 15 marsupial species from 73 localities (Fig. 1, Table 1), with an average of 1.63 species, a maximum of five and a minimum of one species per locality (Fig. 1). These localities account for 117 new records, representing a 53.4% increase from those previously known for MS state (*n* = 219; Table 2). In addition, these new records represent an increase between 96.7% (*Gracilinanusagilis*) to 9.1% (*Chironectesminimus*) of those previously known, with an average increase of 45% (Table 2). The two records of *Cryptonanusagricolai* represent the first for MS state; the single record of *D.aurita* is the first for the Cerrado ecoregion in MS (in a gallery forest of the Paraná River basin); the record of *Cryptonanuschacoensis* is the first for the Chiquitano forest in MS (Table 2); and records of *Marmosarapposa* (Bela Vista, MS), *Monodelphisdomestica* (Porto Murtinho, MS), *Philandercanus* (Caracol, MS), and *Thylamysmacrurus* (Bela Vista, MS) are the southernmost ones in Brazil for these species.

**Table 2.** Number of recorded species per ecoregion (*sensu*Dinerstein et al. 2017) reported for Mato Grosso do Sul state and those previously known from the literature (between parentheses).

Species	Chiquitano forest	Pantanal	Cerrado	Atlantic forest	Total	% increase
*Chironectesminimus*	0 (0)	0 (1)	1 (7)	0 (3)	1 (11)	9.1
*Caluromyslanatus*	0 (0)	0 (1)	0 (2)	0 (2)	0 (5)	
*Caluromysphilander*	0 (0)	0 (0)	1 (5)	0 (0)	1 (5)	20.0
*Cryptonanusagricolai*	0 (0)	0 (0)	1 (0)	1 (0)	2 (0)	
*Cryptonanuschacoensis*	1 (0)	0 (3)	3 (6)	1 (2)	5 (11)	45.5
*Didelphisaurita*	0 (0)	0 (0)	1 (0)	0 (2)	1 (2)	50.0
*Didelphispoecilotis*	0 (2)	2 (2)	17 (26)	5 (11)	24 (41)	58.5
*Gracilinanusagilis*	0 (3)	5 (9)	21 (14)	3 (4)	29 (30)	96.7
*Lutreolinacrassicaudata*	0 (0)	0 (0)	1 (4)	0 (6)	1 (10)	10.0
*Marmosamurina*	0 (1)	1 (1)	4 (10)	2 (6)	7 (18)	38.7
*Marmosarapposa*	1 (2)	0 (0)	9 (12)	0 (0)	10 (14)	71.4
*Marmosopsocellatus*	1 (3)	0 (1)	0 (0)	0 (0)	1 (4)	25.0
*Metachirusmyosuros*	0 (1)	0 (0)	0 (0)	0 (1)	0 (2)	
*Monodelphisdomestica*	2 (5)	2 (7)	7 (4)	0 (0)	11 (16)	68.7
*Monodelphiskunsi*	1 (4)	0 (0)	1 (10)	0 (6)	2 (20)	10.0
*Philandercanus*	0 (2)	2 (5)	2 (3)	0 (0)	4 (10)	40.0
*Thylamysmacrurus*	0 (0)	1 (6)	17 (14)	0 (0)	18 (20)	90.0
Total	6 (20)	13 (37)	86 (120)	12 (43)	117 (219)	53.4
Percentage increase by ecoregion	30.0	35.1	71.6	27.9	53.4	

**Figure 2.**
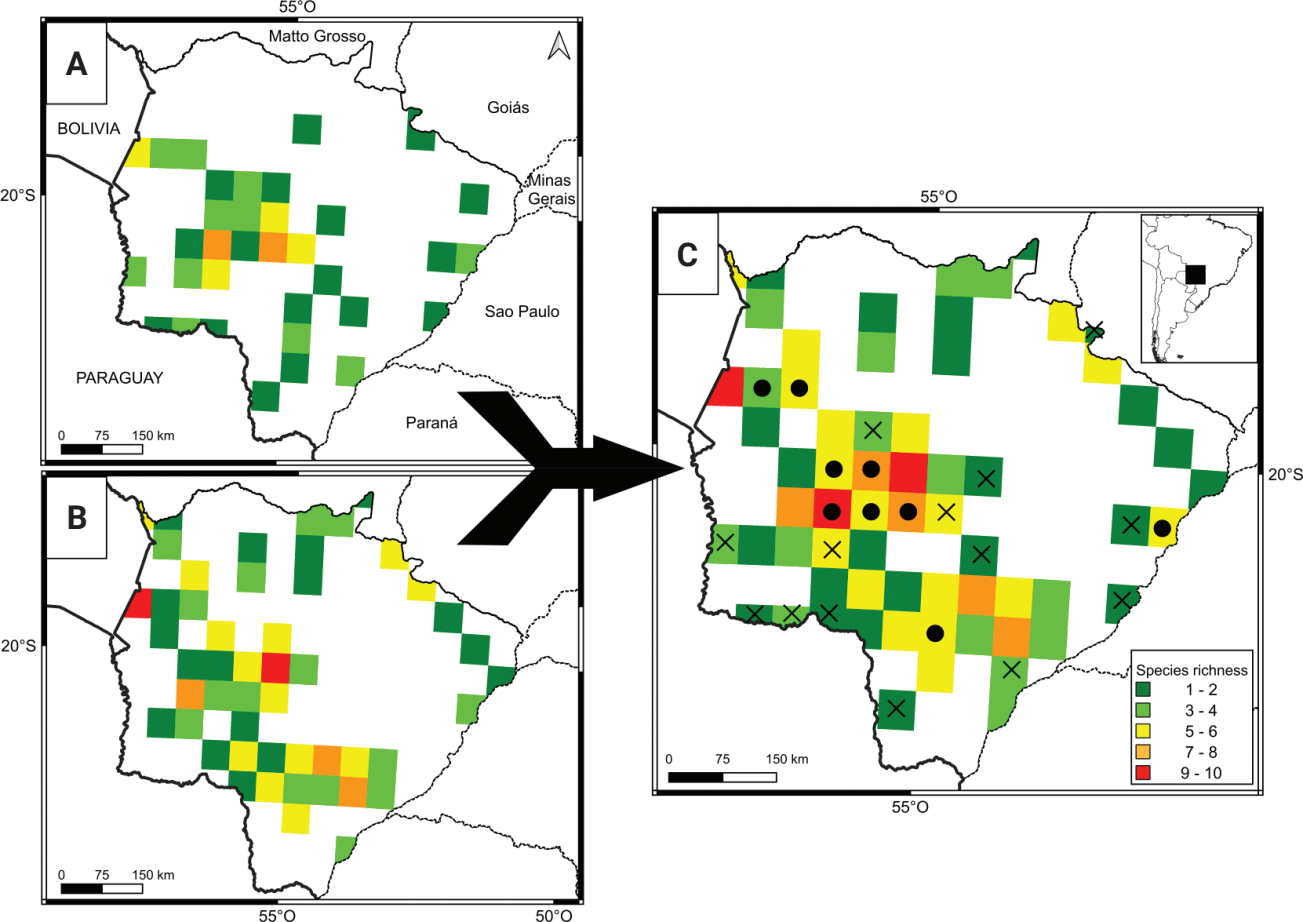
Marsupial species richness in Mato Grosso do Sul state in grids of 0.5° × 0.5° quadrats. **A.** Authors’ data, **B.** Literature data, **C.** Total species richness combining data from this study and previous literature. An X indicates a new record to a given quadrat where no species was previously recorded; a black circle indicates new contributions (i.e., one or more new species recorded) to a previously known quadrat from literature.

With our data, we generated 35 grids in the state (Fig. 2A), with a richness between 7 and 1 species, 13 of them with new records for MS (black X in Fig. 2C). Previous data for marsupials in the state were included in 50 grids (Fig. 2B). In addition, our findings increased the species richness in nine different grids, in some cases incorporating more than five species to the known richness (black dots in Fig. 2C). Our results show an increase in marsupial richness in different regions of MS state, but particularly in the southwest and central regions (Fig. 2C).

**Figure 3.**
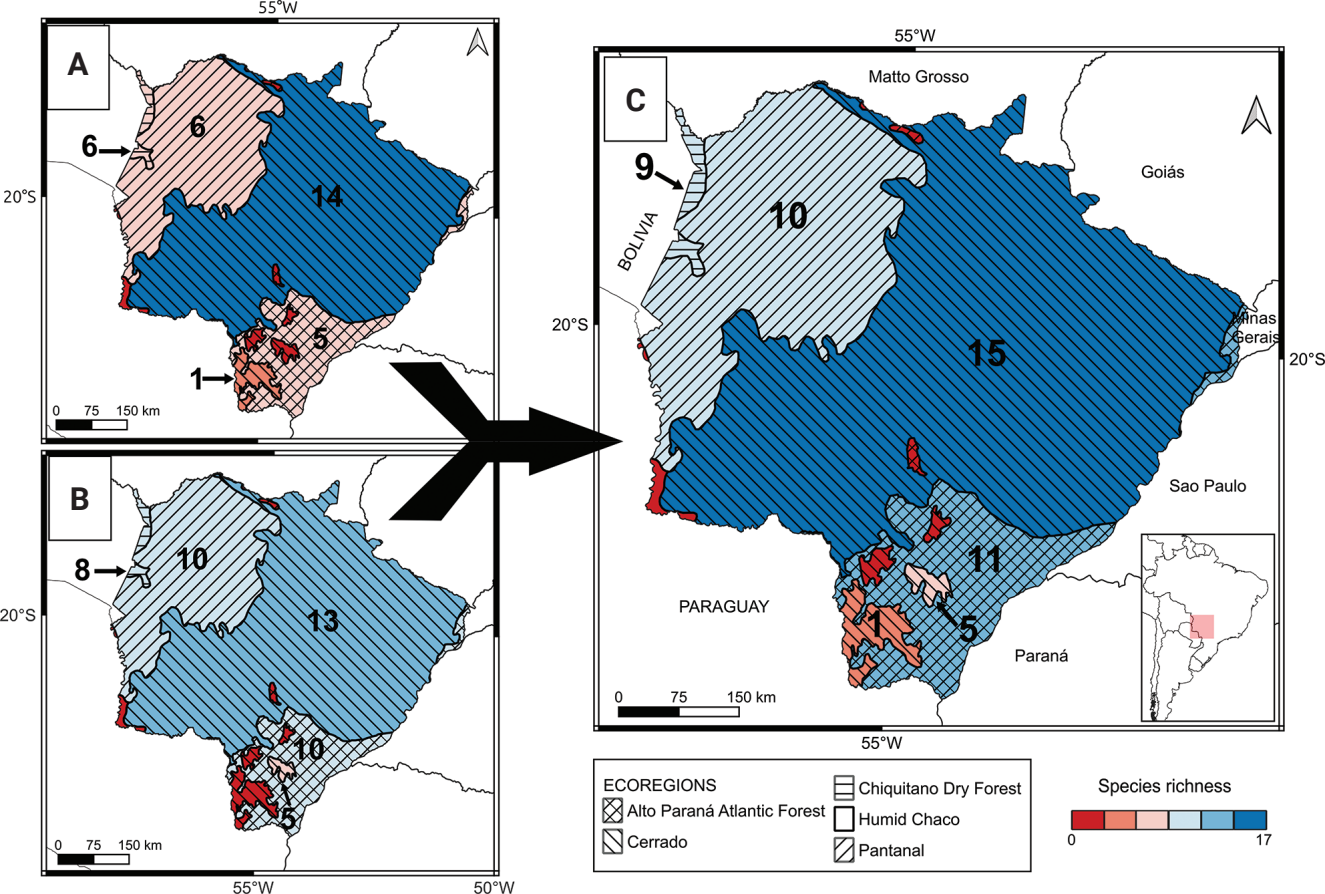
Marsupial species richness by ecoregion in Mato Grosso do Sul state. Different colours show different ecoregion richness, which is also indicated by the number of species. **A**. Authors’ data, **B**. Literature data, **C**. Total species richness combining data from this study and previous literature.

Regarding ecoregional richness, we recorded 14 marsupial species in the Cerrado, six in the Pantanal and the Chiquitano forest, and five in the Atlantic forest (Table 2). No records were made in the small portion of the Humid Chaco, in the extreme southwestern portion of MS (Figs 1, 3). The majority of our records also came from the Cerrado (*n* = 86), with the smallest number (*n* = 6) from the Chiquitano forest (Fig. 1). Our results increased the number of species in the Atlantic forest and Chiquitano forest, with records of *C.agricolai* and *C.chacoensis*, respectively, also increasing the overall number of records in ecoregions by between 71.6% and 27.9%, with the highest number of records in the Cerrado, followed by the Pantanal (Table 2, Fig. 3). Contrary to our prediction, we found more species in the Cerrado ecoregion and similar species richness in the Atlantic forest, Pantanal, and Chiquitano forest, even when combining our data with that of the literature (Fig. 3).

### ﻿Species account

Below, we provide an annotated list of marsupial species from MS state, based on our own records and data from the literature. Regional information for each species is provided when available, as follows: SE: southeast; SW: southwest; NE: northeast; NW: northwest. Specimen collection number from the Mammal Collection at Universidade Federal de Santa Maria is provided for representative records; a full list of UFSM voucher specimens is provided in Table 1. In addition, geographic coordinates, municipality, or locality in MS state are given for some cases, such as when the municipality comprises a large area.

#### ﻿Didelphimorphia

**Didelphidae**

**Caluromyinae**

##### 1) *Caluromyslanatus* (Olfers, 1818)

**Figure 4.**
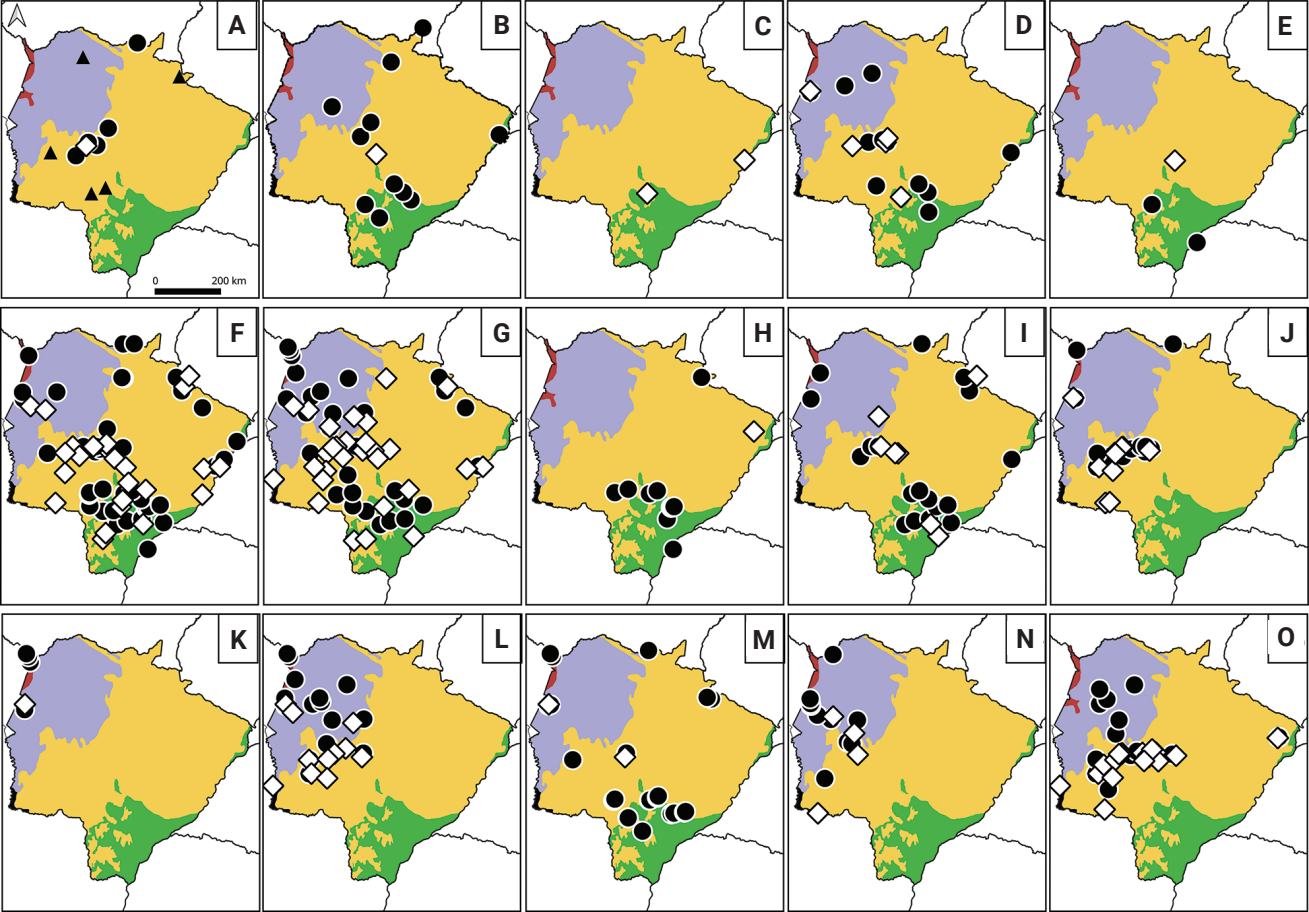
Distribution of marsupial species in Mato Grosso do Sul state, including data from the literature (black circles) and new data from this work (white diamonds). Black triangles were used to show species we did not sample. **A***Caluromysphilander* and *C.lanatus*; **B***Chironectesminimus*; **C***Cryptonanusagricolai*; **D***Cryptonanuschacoensis*; **E***Didelphisaurita*; **F***Didelphispoecilotis*; **G***Gracilinanusagilis*; **H***Lutreolinacrassicaudata*; **I***Marmosamurina*; **J***Marmosarapposa*; **K***Marmosopsocellatus*; **L***Monodelphisdomestica*; **M***Monodelphiskunsi*; **N***Philandercanus*; **O***Thylamysmacrurus*. Ecoregions follow Dinerstein et al. (2017): Alto Parana Atlantic Forest (green); Cerrado (orange); Chiquitano Dry Forest (red); Pantanal (purple); and Dry Chaco (black).

We did not capture this species in the state. This species is known from five localities in MS state, two in the Cerrado, one in the Pantanal, and two in the Atlantic forest (Table 2). The species appears to be rarer than *C.philander* in the state but occurring in different ecoregions (Fig. 4A). Deciduous Atlantic forest should favour its occurrence, such as in the Bodoquena Mountains (Cáceres et al. 2007a), which is a deciduous Atlantic forest relict inserted in the SW Cerrado (Prado and Gibbs 1993), and in Maracaju county, inserted in the main block of Atlantic forest in the state (Carmignotto 2004; W. Hannibal, pers. comm. 26 March 2025). This species was recorded in eastern Paraguay, not so far from the border with MS state (Redford and Eisenberg 1992).

##### 2) *Caluromysphilander* (Linnaeus, 1758)

We recorded this species in a single locality within the Cerrado (Table 2, Fig. 4A), in an urban environment (a woodland square) but close to a gallery forest (< 100 m) of the Aquidauana River. The species was previously known from five records, four from the Cerrado and one from the northern Pantanal (Antunes et al. 2021). The species occurs in the SW Cerrado region, along gallery forests, surrounding the Pantanal in the Paraguay River basin (Fig. 4A) where it appears to be locally common. Occurrence in the NE Cerrado region is expected (Rodrigues 2004), although not registered yet. Occurrence in the southern Pantanal region is also expected, since it is already confirmed in the northern Pantanal (MT state; Aragona and Marinho-Filho 2009). Collection number: UFSM 234 (Aquidauana, MS).

#### ﻿Didelphinae

##### 3) *Chironectesminimus* (Zimmermann, 1780)

Our only record of this species is from a deciduous forest of the Cerrado ecoregion (Fig. 4B). The species was previously known from 11 localities, seven from the Cerrado, three from the Atlantic forest, and one from the Pantanal (Table 2). Field observations of this species are fairly common but are rarely confirmed by captures. A camera-trap study suggests its presence in the Pantanal region (Silveira et al. 2006). Collection number: UFSM 031 (Sidrolândia, MS).

##### 4) *Cryptonanusagricolai* (Moojen, 1943)

**Figure 5.**
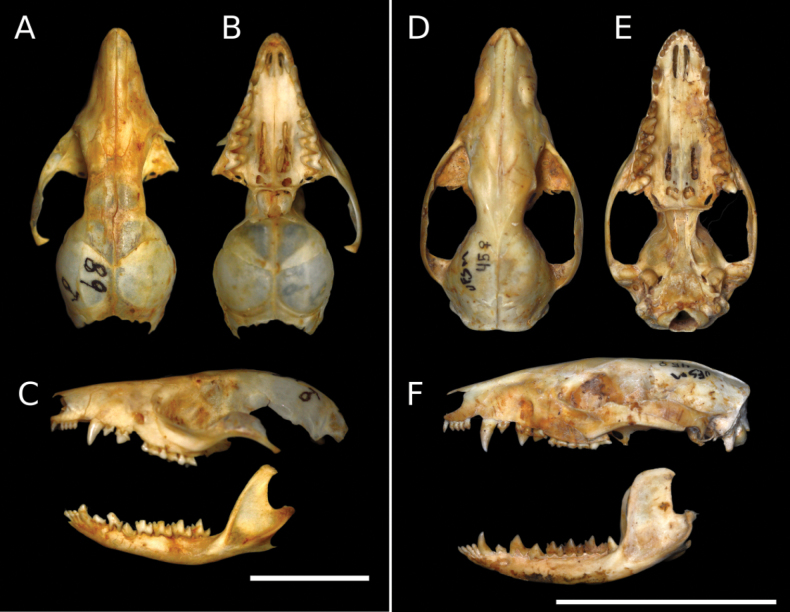
Photographs of the skull and mandible of taxonomic representative marsupial species collected in Mato Grosso do Sul state. **A–C.***Cryptonanusagricolai* (UFSM_089) sampled in Três Lagoas, MS, in July 2003; **D–F.***Didelphispoecilotis* (UFSM_045) sampled in Santa Rita do Pardo, MS, in January 2003. Scale bars: 1 cm (**A–C**); 5 cm (**D–F**).

This species was identified based on the following combination of characters: yellowish ventral fur, small molars, and a complete anterior cingulum on M3 (it is usually incomplete in *C.chacoensis*) (Voss et al. 2005). Apart from the above characteristics, it can be separated from *C.chacoensis* by its larger paracone (notoriously smaller than the metacone in *C.chacoensis*), more developed ectoflexus in M2, and larger protoconid and hypoconid than in *C.chacoensis* (Fig. 5A–C). We recorded this species in two distant localities, one in the east, near the border with São Paulo state (locally in Atlantic forest patches close to the Paraná River) and the other in the south-central part of the state, in the Atlantic forest ecoregion (Fig. 4C). These are the first records for the species in MS and are associated with semi-deciduous forest rather than savanna habitat. Collection numbers: UFSM 019 (Rio Brilhante, MS) and 089 (Estância Figueira, Três Lagoas, MS).

##### 5) *Cryptonanuschacoensis* (Tate, 1931)

We recorded this species in five localities (Table 2, Fig. 4D), three in the Cerrado, one in the Atlantic forest, and one in the Chiquitano forest (Urucum mountains). The species was previously known from 11 localities, six in the Cerrado, three in the Pantanal, and two in the Atlantic forest (Table 2). Carmignotto (2004) reported *Gracilinanuschacoensis* (currently included in the genus *Cryptonanus*) for Reserva Particular do Patrimônio Natural Acurizal at Corumbá, MS, which is an area under the influence of Chiquitano forest. It appears to be common in MS state, particularly in the west. Collection numbers: UFSM 680 (Morro Santa Cruz, Corumbá, MS) and 647 (Fazenda Cachoeirão, Terenos, MS).

##### 6) *Didelphisaurita* (Wied-Neuwied, 1826)

This species is only known from three localities in MS, with a single new record for the Campo Grande municipality (a dead individual run over in a bridge crossing a gallery forest [Anhanduí county]) and two previous records for the Atlantic forest (Table 2, Fig. 4E). The previous records are from (1) the right Paraná River margin, in the border (Naviraí, Porto Caiuá, MS) between Mato Grosso do Sul and Paraná states and the Maracaju county (Carmignotto 2004), both from the Atlantic forest. The species appears to be underrepresented in records from the state, particularly in the Atlantic forest ecoregion, where its presence is anticipated (Cáceres et al. 2016). Due to forest fragmentation, the species might be undergoing an ecological replacement by *D.poecilotis* in the region. No specimens were collected of this species.

##### 7) *Didelphispoecilotis* A. Wagner, 1842

We follow Chemisquy et al. (2025) in recognising this species as different from *D.albiventris* (Fig. 5D–F), which was identified based on the following character combination: talon of P3 clearly separated in two cingula, well separated paracone and metacone, presence of a labial cingulum between the posterior crest of stylar cusp B and the ectoflexus, a large metacone and metastylar area, and well-developed and large anterobasal cingulum. This species was recorded in 24 localities, with 17 of them in the Cerrado, five in the Atlantic forest, and two in the Pantanal (Table 2), representing more than a 58% increase in the species’ known records. The species was previously known from 41 records, mostly from the Cerrado ecoregion (e.g., Antunes et al. 2021), and is widespread in all regions of the state (Carmignotto 2004; Cáceres et al. 2008). It is probably invading more forested habitats due to fragmentation, replacing forest dwellers like *D.aurita* in ecotonal regions (Cerqueira 1985). Collection numbers: UFSM 045 (Fazenda Conquista, Santa Rita do Pardo, MS), 046 (Piraputanga, Aquidauana, MS), and 245 (Fazenda Bela Vista, Nova Alvorada do Sul, MS).

##### 8) *Gracilinanusagilis* (Burmeister, 1854)

We recorded this species in 29 localities, 21 in the Cerrado, five in the Pantanal, and three in the Atlantic forest (Table 2). The species was previously known by 30 records, most of them from Cerrado, and only three records from Chiquitano forest, where we did not record it (Table 2, Fig. 4G). Intensive effort in the Urucum mountains revealed no capture of this species (Hannibal et al. 2017). However, the species occurs in all ecoregions of the state (Carmignotto 2004; Cáceres et al. 2008). Collection numbers for representative localities: UFSM 086 (Três Lagoas, MS), 207 (Dois Irmãos do Buriti, MS), 252 (Chapadão do Sul, MS), 371 (Corguinho, MS), 469 (Amambai, MS), 557 (Fazenda Xaraés, Corumbá, MS), and 613 (P.E. Várzeas do Rio Ivinhema, Ivinhema, MS).

##### 9) *Lutreolinacrassicaudata* (Desmarest, 1804)

We recorded this species in only one locality in the Cerrado (Table 2, Fig. 4H), but it was previously known from ten localities, eight from the Atlantic forest and two from the Cerrado (Table 2). The species is known from few records in the state, and its occurrence is probably regionally underestimated, particularly in the NE Cerrado. Interestingly, there is no reliable record in the Pantanal, which could represent an ideal habitat for the species based on its known biology (Redford and Eisenberg 1992). Collection number: UFSM 326 (Inocência, MS).

##### 10) *Marmosamurina* (Linnaeus, 1758)

The taxonomy of *M.murina* was studied by Rossi (2005), Faria et al. (2013), and Voss et al. (2014). Our specimens from MS differ in some craniodental features from the species description of Voss and Jansa (2009), which include the shape of the nasals, the presence of a well-developed, thin dorsal branch of the premaxillary, the shape of the yugal and its contact with the lacrimal, and the overall shape of dp3 and m1. However, pending a thorough revision and comparisons, we tentatively assigned our specimens to *M.murina*. We recorded this species seven times, distributed in the Cerrado (*n* = 4), the Atlantic forest (*n* = 2), and the Pantanal (*n* = 1) ecoregions; the literature reported similar records across ecoregions, plus one record in the Chiquitano forest (Table 2, Fig. 4I). This species occurs in all ecoregions of the state, including the Pantanal (Carmignotto 2004), except for the non-sampled Humid Chaco in the west. It appears to be related to forest patches overall and to gallery forest when in the Cerrado (Hannibal and Cáceres 2010). Intensive efforts in Chiquitano forest resulted in no captures of this species (Cáceres et al. 2011a), but Hannibal et al. (2017) reported a captured specimen that is apparently not present in any mammal collection as a voucher. Collection numbers: UFSM 325 (Costa Rica, MS), 372 (Fazenda Santana, Aquidauana, MS), 612 (Naviraí, P.E. Várzeas do Rio Ivinhema, MS), 634 (Terenos, MS), and 903 (Novo Horizonte do Sul, MS).

##### 11) *Marmosarapposa* Thomas, 1899

We follow Voss et al. (2020) in recognising this species as different from *M.constantiae* (Thomas, 1904) by the following dental characters: well-developed postprotocrista that extends beyond the base of the metacone as a cingulum, and presence of a well-developed posterior cingulid at the base of the hypoconid. We recorded this species in 10 localities, nine of them in the Cerrado and one in the Chiquitano forest (Table 2, Fig. 4J). The species was previously known from 14 localities, 12 of them from the Cerrado and two from the Chiquitano forest (Table 2). It is a species with a western distribution in the state, which includes the SW Cerrado (Cáceres et al. 2007a; Hannibal and Cáceres 2010), the Pantanal (Carmignotto 2004), and the Chiquitano forest (Cáceres et al. 2011a), all within the Paraguay River basin. However, the presence of this species is indicated also at Campo Grande (Vieira 1955), a locality in the Paraná River basin (covered with woodland savanna), just on the border with the Paraguay River basin (SW Cerrado). Our field efforts indicate that this species is rare and mostly absent in the NE Cerrado and in the Atlantic forest (and even in eastern Paraguay; De la Sancha et al. 2011). The congeneric *M.paraguayana* (Tate, 1931) could be present in the Atlantic forest of MS state, which is supported by its presence in the Atlantic forest of eastern Paraguay (De la Sancha et al. 2011), in the border of MS state. Representative collection numbers: UFSM 013 (Fazenda Princesinha, Bonito, MS), 628 (Anastácio, MS), and 789 (Morro Santa Cruz, Corumbá, MS).

##### 12) *Marmosopsocellatus* (Tate, 1931)

Formerly considered as *M.dorothea*, the species is restricted to the Chiquitano ecoregion of MS to which we added a single record (Carmignotto 2004; Cáceres et al. 2007b) (Table 2, Fig. 4K). All previous records are from the Chiquitano forest, particularly the Amolar (north) and Urucum (south) uplands in the NW of the state. The species lives in deciduous forest habitats of this region (Cáceres et al. 2011a). Collection numbers: UFSM 351 and 605 (Morro Santa Cruz, Corumbá, MS).

##### 13) *Metachirusmyosuros* (Temminck, 1824)

Despite intensive trapping, we did not record this species for MS, and it is only known from two previous records, one for the SE Atlantic forest (12 km N of Dourados) and the other for the Chiquitano forest, restricted to the Santa Cruz mountain, Urucum (Gardner, 2008) (Table 2). The presence of this species in the state deserves confirmation, since there are no voucher specimens known (Tomás et al. 2017), and Carmignotto (2004) did not record this species in MS state after extensively reviewing museum collections for her PhD thesis. However, we expect the occurrence of *Metachirus* for MS state, as it occurs in adjacent eastern Paraguay southward (Redford and Eisenberg 1992; Bonvicino et al. 2023) and in deciduous forests of Mato Grosso state northward (Brandão et al. 2019), which are regions contiguous with MS state (Carmignotto 2004). Particularly in the Atlantic forest of MS state, conservation issues (see section below) make the sampling of more sensitive species to human disturbance difficult, which we expect to be the case of *M.myosuros*.

##### 14) *Monodelphisdomestica* (Wagner, 1842)

We recorded this species mostly in the SW Cerrado (*n* = 7), but also in the Pantanal (*n* = 2) and the Chiquitano forest (*n* = 2) (Table 2, Fig. 4L). The species is also known from several previous records mostly in the Pantanal (Vieira 1955; Carmignotto 2004; Cáceres et al. 2007a, 2010; Aragona and Marinho-Filho 2009; Andreazzi et al. 2011) but also in the Chiquitano forest (Cáceres et al. 2011a). Overall, this species was recorded in the west of MS state. Records from the south of Goiás indicate its occurrence in the NE region of the Cerrado in MS (Rodrigues 2004; Carmignotto 2004), although we did not capture this species in this area (Fig. 4L). There is evidence that this species is affected by forest fragmentation of Cerrado vegetation (Cáceres et al. 2010; Melo et al. 2022), which is a possible reason for its absence in the NE Cerrado region of MS state, an area more affected by habitat fragmentation than the west (SW Cerrado; Machado et al. 2004). Representative collection numbers: UFSM 010 (Fazenda Princesinha, Bonito, MS), 029 (Fazenda Califórnia, Bodoquena, MS), 373 (Fazenda Santana, Aquidauana, MS), 559 (Albuquerque, Corumbá, MS), and 717 (Morro Santa Cruz, Corumbá, MS).

##### 15) *Monodelphiskunsi* Pine, 1975

We recorded this species in specific localities of SW Cerrado and the Chiquitano forest, but it has also been captured in the same ecoregions and in the Atlantic forest by other researchers (Table 2, Fig. 4M). The species is widely distributed in MS state, occurring in all main ecoregions but it was not recorded in the Pantanal (Fig. 4M; Cáceres et al. 2010; Hannibal et al. 2012). In the Atlantic forest, it occurs in ecotonal areas with Cerrado vegetation (Hannibal et al. 2012). In upland regions of forest-savanna mosaics (at Urucum mountains, covered by Chiquitano forest), this species tends to occur in more open vegetation such as upland grassland (Cáceres et al. 2011a). Since this species is only captured with pitfall traps (Cáceres et al. 2011b), its absence in the Pantanal—where it is expected—may be due to sampling limitations. Collection numbers: UFSM 167 (Dois Irmãos do Buriti, MS) and 696 (Morro Santa Cruz, Corumbá, MS).

##### 16) *Philandercanus* (Osgood, 1913)

We recorded this species twice in SW Cerrado and in the Pantanal ecoregions (*n* = 4 records), with a southern record close to the border with Paraguay and the Humid Chaco ecoregion (Table 2, Fig. 4N). This species is restricted to the western region of the state, close to the Paraguay River and tributaries, mostly in the Pantanal wetlands and surrounding savanna, and also in deciduous western forests (10 previous records; e.g., Vieira 1955; Carmignotto 2004; Herrera et al. 2007; Cáceres et al. 2011a). Its occurrence in the SW Cerrado appears to be in ecotonal areas with Pantanal, Humid Chaco, and Chiquitano forest ecoregions (Fig. 4N). It has also been recorded northward in econotal areas of the Cerrado, usually in humid forests near water sources (Aragona and Marinho-Filho 2009). Taxonomic remark: the southern and easternmost record of *Philander* in MS state (Mundo Novo municipality; Gardner 2008) is thought to be *P.quica* (opossum), aligned with its presence in the Atlantic forest of adjacent eastern Paraguay (Voss et al. 2018). This record of Mundo Novo is problematic because it is assigned as from Rio Grande do Sul (RS) state in Brazil, not Mato Grosso do Sul state, which we think was a mistake, since the locality point in the map (in Gardner 2008) is clearly referring to Mundo Novo, MS (not RS). Therefore, we excluded this locality from our map, not considering *P.quica* for MS state. Only capture/release or photographs (Table 1, Fig. 4N).

##### 17) *Thylamysmacrurus* (Olfers, 1818)

Most of our records of this species are from SW Cerrado (*n* = 17), with a single record in the southernmost extension of the Pantanal (Porto Murtinho), near a small portion of Humid Chaco (Fig. 4O). This species occurs predominantly in the SW Cerrado (20 previous records; Carmignotto 2004; Cáceres et al. 2007a, 2010) and in the southern Pantanal (6 previous records; Andreazzi et al. 2011), but not in the Chiquitano forest (Fig. 4O). *Thylamysmacrurus* was previously known from the Paraná River basin at Campo Grande (Vieira 1955), and posteriorly confirmed by us east of Campo Grande city (Fig. 4O). This species is locally abundant in larger forest fragments of western woodland savanna (SW Cerrado; Cáceres et al. 2010), but is also common in seasonal, dry forests of the Bodoquena mountains (Cáceres et al. 2007c). Intensive efforts did not reveal its presence in the Chiquitano forest (Cáceres et al. 2011a; Hannibal et al. 2017). Outside MS to the south, the species is found in the Humid Chaco in Paraguay (Giarla et al. 2010; Astúa et al. 2023), always following the Paraguay River margin and left tributaries. Collection numbers: UFSM 005 (Fazenda Princesinha, Bonito, MS), 035 (Faz. Santa Terezinha, Bonito, MS), 049 (Piraputanga, Aquidauana, MS), 487 (Inocência, MS), 554 (Fazenda Sossego, Campo Grande, MS), 631 (Terenos, MS), 636 (Miranda, MS), and 359 (Dois Irmãos do Buriti, MS).

### ﻿Species distribution, species limits, and climate

Based on species composition by ecoregion, we established distribution limits for more range-restricted species in MS, following congruent biogeographic patterns among subsets of species pools (for example, species in Fig. 4A, J, L, N, O are restricted to the west of the state). These main species limits do not necessarily follow ecoregions’ limits but could extend little beyond them, just because of the influence of transitional areas between ecoregions and the capacity of some species to go slightly beyond the vegetation type they commonly inhabit (such as the cases of *P.canus* going outside the Pantanal toward the south and *T.macrurus* going outside the SW Cerrado toward the east; Fig. 4N, O). Species from eastern ecoregions (like the Atlantic forest and NE Cerrado) are mostly limited to the plateau of Maracaju, not expanding to the Paraguay basin in the west, like *C.agricolai*, *D.aurita*, and *L.crassicaudata* (Fig. 4C, E, H). Another species boundary is found for *M.ocellatus*, which is confined to the Chiquitano forest in northwestern MS, not crossing the Paraguay River to the east (Fig. 4K).

**Figure 6.**
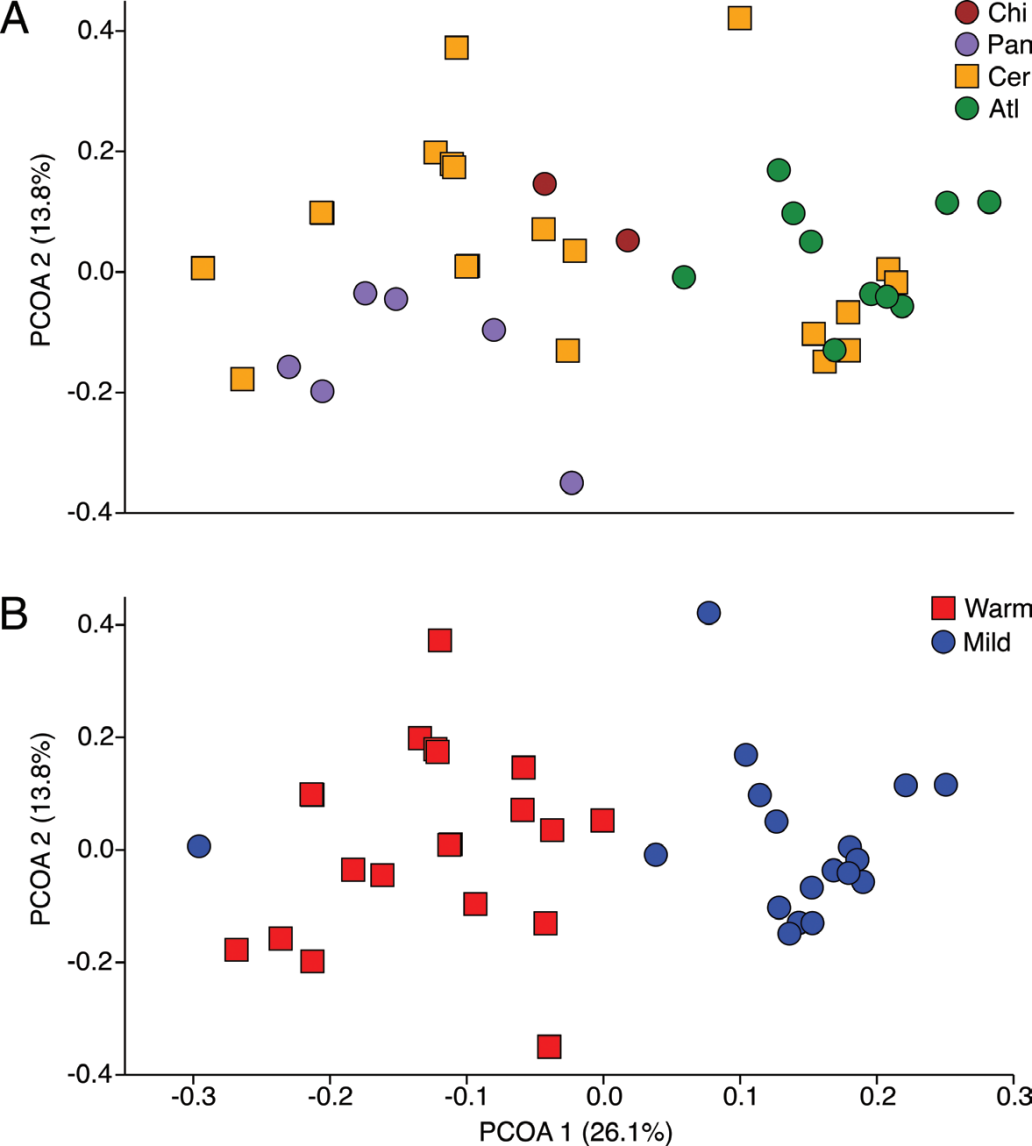
Turnover of marsupial species composition in Mato Grosso do Sul state, based on our data and those from the literature. **A**. Gradient of community variation according to a PCoA analysis, showing ecoregions with different colours (Atl = Atlantic forest; Cer = Cerrado; Chi = Chiquitano forest; Pan = Pantanal). **B**. Gradient of community variation according to a PCo analysis, showing mild (< 23.5 °C, in blue) and warm (> 23.5 °C, in red) localities.

**Figure 7.**
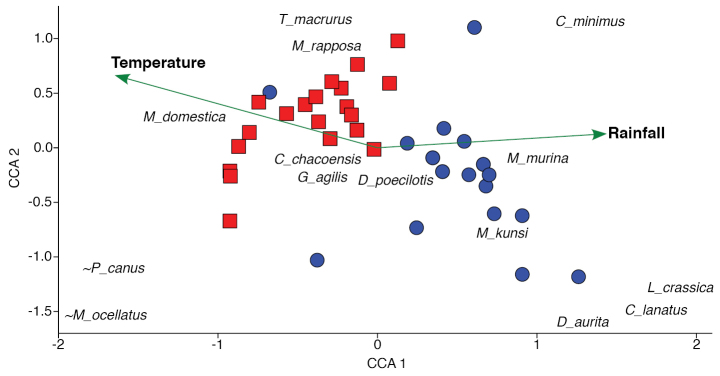
Turnover of marsupial species composition in Mato Grosso do Sul state, based on our data and those from literature. Gradient of community variation according to a CCA analysis, showing climatic (green) vectors, colder/mild (blue) and warmer (red) localities, and marsupial species (initials for genus) that contributed to the community turnover. *Marmosopsocellatus* and *Philandercanus* are indicated with a “~” to highlight that their (extreme) positions in the gradient (axis 1) were shortened in order to appear in the plot.

We selected 41 quadrats distributed across MS state, all of them containing at least three species, and found a strong climatic gradient, which was correlated with the species composition in such quadrats. This was particularly important for the mean annual temperature (Fig. 6B) for which quadrats varied from 21.5 °C to 26.3 °C (~ 5 °C of difference between the extremes). Therefore, we found a strong east-west gradient of faunal turnover which follows temperature (r = -0.64; p < 0.0001 for PC1) and rainfall (r = +0.51; p < 0.001 for PC1) gradients. The uplands of Bodoquena Mountains are an exception in the west of MS state, where a mild climate could be found (note the outlier area in blue following the negative side of axis 1 in Fig. 6B, where we sampled species like *T.macrurus*, *M.domestica*, and *M.rapposa* (Fig. 7)). Overall, the west of the state (where SW Cerrado, Pantanal, and Chiquitano forest occur) is drier and warmer than the east (where NE Cerrado and Atlantic forest occur). Thus, quadrats from the SW Cerrado and Pantanal show greater similarity to each other (negative values of axis 1), as well as NE Cerrado and Atlantic forest (positive values of axis 1), because of the similarity in species composition among them, according to the multivariate PCoA analysis (Fig. 6A) based on 17 marsupial species. PCoA axis 1 was responsible for 26.1% of the variation and was related to the species compositional differences related to the Atlantic forest and NE Cerrado in the east of the state (determined mainly by the presences of *D.aurita*, *L.crassicaudata*, *C.agricolai*, and *M.murina*) and to the SW Cerrado, Pantanal, and Chiquitano forest in the west (determined mainly by the presences of *M.ocellatus*, *P.canus*, *M.domestica*, *T.macrurus*, and *M.rapposa*) at the other extreme (Fig. 7). Therefore, there is a continuum of marsupial community change from the Atlantic forest toward the Pantanal and Chiquitano forest, representing a predominantly southeast-northwest gradient.

## ﻿Discussion

### ﻿Sampling effort

Across MS state, field sampling has been concentrated mostly in the west, with limited efforts in the east and north (e.g., Fig. 1). We think that this trend is mostly driven by the Pantanal wetlands and the adjacent Cerrado in the west, which has attracted many researchers and graduate students (see e.g., Antunes et al. 2021). However, we expect that new marsupial species will be recorded in the Chiquitano forest, NE Cerrado, and in the Atlantic forest. In recent years, new mammal species with Amazonian affinities have been recorded in the Chiquitano forest and Pantanal wetlands, like the marsupial *Glironiavenusta* Thomas, 1912 in the south of Mato Grosso state (Rossi et al. 2010) and the spectral bat *Vampyrumspectrum* (Linnaeus, 1758) in the Pantanal (Silveira et al. 2011). Also, an inventory in the Emas National Park (Rodrigues 2004) just close to the northern border of MS state suggests that other new species could be added to the NE Cerrado with more collections (e.g., *Thylamysvelutinus* (Wagner, 1842); Rodrigues 2004; Carmignotto 2004).

Our work also revealed some interesting distributional patterns like those of *D.aurita*, *L.crassicaudata*, and *C.agricolai* in the Atlantic forest south of MS state. However, we feel we were unable to sample other probable species occurring in adjacent eastern Paraguay such as *M.paraguayana* and even *D.aurita*, which was rare in our sample. The same is true for *P.quica*, which might occur in the Atlantic forest of MS state given its presence in adjacent, eastern areas of Paraguay (Voss et al. 2018; Astúa et al. 2023). Indeed, in eastern Paraguay some marsupial species typical of the Atlantic forest have been reported, like *D.aurita*, *M.paraguayana*, and *M.myosuros* (Bonvicino et al. 2023), together with common Atlantic forest rodent species such as *Euryoryzomysrussatus* (Wagner, 1848), *Juliomyspictipes* (Osgood, 1933), *Sooretamysangouya* (Fischer, 1814), and *Thaptomysnigrita* (Lichtenstein, 1829) (Redford and Eisenberg 1992; De la Sancha et al. 2009, 2011). Therefore, a more diversified marsupial fauna is expected for the Atlantic forest of MS (like the “Refúgio Biológico de Maracaju”, in Mundo Novo, MS), based on the diversity reported for adjacent Paraguay (but see ‘Conservation issues’ below).

### ﻿Biogeographical affinities

The diagonal of open areas present in central Brazil encompassing the Caatinga, Cerrado and Pantanal ecoregions (Ab’Saber 1977) mostly drives the species composition of this region. Subdivisions of this large area are reasonable, like the five ones recognised by Carmignotto (2004), which segregate our SW Cerrado from other savanna areas northward. Following that proposal, our NE Cerrado ecoregion would be linked to the Cerrado of Goiás, São Paulo, and Minas Gerais states, sharing some species. In addition, we propose that our SW Cerrado plus the southern Pantanal would create an additional biogeographical region, linked to the Cerrado of central Paraguay (sharing species like *M.rapposa*, *M.domestica*, and *T.macrurus*, with the absence of common Cerrado species like *M.constantiae* and *T.velutinus* which have eastern and northern distributions, respectively; Redford and Eisenberg 1992; Cáceres et al. 2007c). Indeed, the species composition of the SW Cerrado of MS has a Chacoan influence, different from the NE Cerrado, with an Atlantic influence (Veloso et al. 1991; Hannibal et al. 2017; this study). The recent recognition of *Sapajuscay* (Illiger, 1815), a southern capuchin monkey from the SE Cerrado and Pantanal, reinforces this proposal (Lynch Alfaro et al. 2012). In the same way, the rodent *Thrichomysfosteri* Thomas, 1903 has a similar distribution, occurring in SW Cerrado and southern Pantanal of MS and Paraguay (D`Elía and Myers 2014). We also expect that new species will be found in the SW Cerrado, such as the case of a new species of *Akodon* found recently (Brandão et al. 2021). Furthermore, the Chaco influences the Pantanal and Chiquitano ecoregions (Veloso et al. 1991), sharing species like *C.chacoensis*, *M.rapposa*, *P.canus*, and *Thylamys* as a genus (Hannibal et al. 2017). An influence of the main block of Atlantic forest from eastern Brazil is expected for the SE Atlantic forest region in MS state. This western Atlantic forest is expected to harbour a mixed community composition with several marsupial species, mainly due to a mixed vegetation of forest and savanna vegetation types (Stevens et al. 2004).

### ﻿Marsupial community turnover and climate

We found a strong correlation between climatic variables and marsupial turnover. Indeed, we identified a marsupial community turnover from east to west, and more precisely from the southeast (Atlantic forest) to the northwest (Chiquitano forest) (Fig. 6). One of the most common species in MS state, *Gracilinanusagilis*, is rare in Chiquitano forest, while *M.ocellatus* only occurs in that ecoregion within MS state (see Cáceres et al. 2007b, 2011a). Overall, MS state could be divided in two portions in an east to west orientation, following the arrangement of the two large river basins, Paraná and Paraguay, with the ridges of Maracaju plateau as an ecoregional and geographic divider. In fact, we found *D.aurita*, *L.crassicaudata*, and *C.agricolai* occurring in the eastern portion of MS state in the Paraná River basin, having a strong Atlantic influence in terms of faunal elements (Fig. 7). Contrary to this pattern, we mostly found *M.ocellatus*, *P.canus*, *M.domestica*, *T.macrurus*, and *M.rapposa* occurring in the western portion of the state, in the Paraguay River basin, with strong Chacoan and Amazonian influences. The Chacoan influence is suggested by shared species between Chaco and the adjacent Cerrado, like the marsupials *T.macrurus* and *M.rapposa* (Giarla et al. 2010; Voss et al. 2020) and the rodent *T.fosteri* (D`Elía and Myers 2014). In addition, the major influence of Amazonian elements in this central-western Brazilian region was previously reported in a phylogeographic study involving small mammals (Costa 2003). Indeed, species occurring in the west of MS, like *P.canus* and *M.ocellatus*, have broader distributions that extend northward, reaching the western Amazon basin (Voss et al. 2018; Bonvicino et al. 2023).

### ﻿Conservation issues

Despite these biogeographical considerations based on marsupial occurrence in MS state, several species were not found in many places due to strong anthropogenic disturbances (Fiaschi and Pirani 2009) that have probably led to species becoming locally extinct. This is a reality in MS state, where many areas, especially towards the east and south, are highly fragmented (Machado et al. 2004) due to cattle ranching and (forestry and soybean) plantations. Inventories in large, undisturbed remnants could reveal a still unknown fauna for this western Brazilian state, mainly using a combination of sampling techniques (Cáceres et al. 2011b).

## ﻿Conclusions

Mato Grosso do Sul state is unique because of the presence of four different biogeographical influences on its fauna, which guide diversity in two forested and humid (Amazonian and Atlantic) and two shrubland and dry (Chaco and Cerrado) ecoregions. This condition provides a peculiar, regional faunal composition of small mammals that exhibits a strong turnover across the state. Our study adds to the knowledge of the mammals of MS state, giving support to the biogeographic patterns described above, based on its marsupial fauna. We believe this biotic pattern is valid for other systematic groups of fauna, especially rodents, bats, and birds, which could be corroborated in further studies.

## ﻿Additional information

### Author ORCIDs

Nilton C. Cáceres https://orcid.org/0000-0003-4904-0604

Geruza L. Melo https://orcid.org/0000-0002-2384-3786

Jonas Sponchiado https://orcid.org/0000-0002-1267-1763

Gabriel M. Martin https://orcid.org/0000-0003-2909-0444

### Data availability

All of the data that support the findings of this study are available in the main text or Supplementary Information.

## Supplementary materials

Supplementary material 1 List of localities sampled in Mato Grosso do Sul state Data type docx Explanation note List of localities sampled in Mato Grosso do Sul state with their geographical coordinates and local species richness. Species sampled are shown per locality (v = voucher; r = released). Number of days sampled within each area is shown in brackets. This file contains the localities sampled by us in MS state, Brazil, providing geographical coordinates, species sampled, ecoregion, and capture effort for each one. https://binary.pensoft.net/file/1359256This dataset is made available under the Open Database License (http://opendatacommons.org/licenses/odbl/1.0/). The Open Database License (ODbL) is a license agreement intended to allow users to freely share, modify, and use this Dataset while maintaining this same freedom for others, provided that the original source and author(s) are credited.Nilton C. Cáceres, Geruza L. Melo, Jonas Sponchiado, Gabriel M. Martin
