# Supplementary material for: ﻿Marsupials (Didelphidae, Mammalia) of Mato Grosso do Sul state (Brazil): taxonomic accounts, species richness, and biogeography
Source: Zookeys. 2025 Jun 23;1243:1–27. doi: 10.3897/zookeys.1243.141601 (PMC12238999; doi:10.3897/zookeys.1243.141601)
Supplement: Supplementary material 1 — List of localities sampled in Mato Grosso do Sul state [file zookeys-1243-001_article-141601__-s001.docx]

**Supplementary Material S1**

List of localities sampled in Mato Grosso do Sul state, Brazil, with their geographical coordinates and local species richness. Species sampled are shown per locality (v = voucher; r = released in the field; ph = with photo). UFSM (Universidade Federal de Santa Maria) museum (Mammal Collection) voucher number and sex (M or F) are provided; when the specimen was deposited in other museum, we inform it by using respective acronym [UFMT (Universidade Federal do Mato Grosso); MHNCI (Museu de História Natural Capão da Imbuia)]. Number of days sampled within each locality is shown in brackets.

| **Locality name and county** | **Lat / Long** | **Altitud (a.s.l.) / Ecoregion** | **Sampled species** | **Museum voucher** | **Sampling effort /day** |
| --- | --- | --- | --- | --- | --- |
| 1. Morro Santa Cruz - Corumbá | 19ø10'13"S 57ø36'59"W | 505 m  Chiquitano F. | C. chacoensis (v)  M. ocellatus (v)  M. kunsi (v)  M. rapposa (v)  M. domestica (v) | 267 (F)  111 (F)  696 (M)  789 (M)  717 (F) | 9 transect lines (8 pitfall traps per line) [60] |
| 2. Albuquerque - Corumbá | 19ø23'50"S 57ø23'55"W | 166 m  Chiquitano F. | M. domestica (v)  G. agilis (r)  D. poecilotis (r) | 559 (M)  -  - | 5 transect lines (13 live traps per line) [4] |
| 3. Fazenda Xaraés - Corumbá | 19ø29'32"S 56ø57'13"W | 94 m  Pantanal | G. agilis (v)  P. canus (r) (ph)  D. poecilotis (r) | 557 (F)  -  - | 5 transect lines (13 live traps per line) [4] |
| 4. Fazenda Caiman – Miranda | 19ø56'14"S 56ø18'35"W | 127 m  Pantanal | G. agilis (r)  P. canus (r) | -  - | 5 transect lines (13 live traps per line) [4] |
| 5. Fazenda Santana - Aquidauana | 19ø37'20"S 55ø36'16"W | 123 m  Pantanal | M. domestica (v)  M. murina (v)  G. agilis (r) | 373 (M)  372 (-)  - | 2 transect lines (30 live traps per line)  1 transect lines (6 pitfall traps per line) [4] |
| 6. Fazenda Rodeio - Corguinho | 19ø45'57"S 55ø13'34"W | 334 m  Cerrado | G. agilis (v) | 371 (F) | 3 transect lines (30 live traps per line)  2 transect lines (6 pitfall traps per line) [4] |
| 7. Fazenda Pouso Frio – Chapadão do Sul | 18ø39'46"S 52ø54'57"W | 834 m  Cerrado | G. agilis (v)  D. poecilotis (r) | 252 (F)  - | 5 transect lines (20 live traps per line) [4] |
| 8. Fazenda Santo Antônio - Costa Rica | 18ø21'23"S 52ø47'38"W | 807 m  Cerrado | M. murina (v)  D. poecilotis (r) | 325 (-)  - | 5 transect lines (20 live traps per line) [4] |
| 9. Fazenda Lindos Campos - Inocência | 19ø49'24"S  51ø32'49"W | 426 m  Cerrado | L. crassicaudata (v)  T. macrurus (v) | 326 (F)  487 (F) | 5 transect lines (12 live traps per line) [4] |
| 10. Porto Conceição - Porto Murtinho | 21ø27'55"S 57ø55'04"W | 86 m  Pantanal | G. agilis (r)  T. macrurus (r)  M. domestica (r) (ph) | -  -  - | 5 transect lines (13 live traps per line) [4] |
| 11. Fazenda Califórnia - Bodoquena | 20ø42'19"S 56ø52'13"W | 470 m  Cerrado | M. domestica (v) | 029 (M) | 2 transect lines (15 live traps per line) [6] |
| 12. Fazenda Princesinha - Bonito | 21ø05'04"S 56ø47'02"W | 558 m  Cerrado | M. domestica (v) (ph)  M. rapposa (v)  T. macrurus (v) | 010 (F)  006 (F)  005 (M) | 3 transect lines (15 live traps per line) [5] |
| 13. Fazenda Santa Tereza - Bonito | 21ø04'25"S 56ø43'05"W | 497 m  Cerrado | G. agilis (r) | - | 5 transect lines (13 live traps per line) [4] |
| 14. Fazenda Santa Terezinha - Bonito | 20ø50'02"S 56ø37'59"W | 615 m  Cerrado | T. macrurus (v) | 035 (M) | 1 transect line (30 live traps per line) [3] |
| 15. Recanto Rio da Prata - Jardim | 21ø26'01"S 56ø26'40"W | 281 m  Cerrado | G. agilis (r) | - | 5 transect lines (13 live traps per line) [4] |
| 16. Fazenda Santa Maria - Bonito | 20ø40'10"S 56ø19'43"W | 184 m  Cerrado | M. rapposa (r) | - | 2 transect lines (10 live traps per line) [20] |
| 17. Fazenda Borboleta - Miranda | 20ø31'00"S 56ø11'16"W | 194 m  Cerrado | G. agilis (r) (ph)  T. macrurus (r) (ph) | -  - | 5 transect lines (13 live traps per line) [4] |
| 18. Fazenda Campo Alegre - Anastácio | 20ø29'35"S 56ø05'52"W | 223 m  Cerrado | M. rapposa (v) (ph)  T. macrurus (v) | 628 (M)  UFMT | 2 transect lines (10 live traps per line) [20] |
| 19. UEMS - Aquidauana | 20ø20'24"S  55ø47'58"W | 214 m  Cerrado | G. agilis (v)  M. domestica (v) | 220 (M)  040 (F) | 1 transect line (15 live traps per line) [4] |
| 20. Centre square - Aquidauana | 20ø28'33"S  55ø48'00"W | 149 m  Cerrado | C. philander (v) | 234 (F) | accidental capture |
| 21. Piraputanga - Aquidauana | 20ø27'16"S 55ø29'53"W | 198 m  Cerrado | D. poecilotis (v)  M. murina (v)  T. macrurus (v) | 046 (-)  536 (F)  049 (F) | 1 transect line (20 live traps per line) [20] |
| 22. Fazenda Santa Helena - Dois Irmãos do Buriti | -20.5201  -55.329171 | 315 m  Cerrado | C. chacoensis (v)  T. macrurus (v) | 477 (F)  678 (M) | 02 trap grids (100 live traps per grid)  08 transect lines (10 pitfall traps per line) [20] |
| 23. Fazenda São Cristóvão - Dois Irmãos do Buriti | -20.562517  -55.304065 | 325 m  Cerrado | G. agilis (v)  M. domestica (r)  M. rapposa (v)  M. kunsi (v)  T. macrurus (v) | 207 (M)  -  534 (M)  167 (M)  359 (M) | 05 trap grids (100 live traps per grid)  08 transect lines (10 pitfall traps per line) [20] |
| 24. Fazenda Cachoeirão - Terenos | 20ø24'13"S 55ø16'57"W | 302 m  Cerrado | C. chacoensis (v) | 647 (M) | 02 trap grids (100 live traps per grid)  08 transect lines (10 pitfall traps per line) [20] |
| 25. Fazenda Primavera - Terenos | 20ø25'33"S 55ø06'11"W | 308 m  Cerrado | T. macrurus (v) | 631 (F) | 2 transect lines (10 live traps per line) [20] |
| 26. Fazenda Sucuri - Terenos | 20ø37'26"S 54ø58'46"W | 267 m  Cerrado | M. murina (v) | 634 (F) | 2 transect lines (10 live traps per line) [20] |
| 27. Fazenda Serrinha - Sidrolândia | 20ø49'58"S 54ø51'48"W | 444 m  Cerrado | C. minimus (v) | 031 (F) | accidental capture |
| 28. Fazenda Nova Esperança - Sidrolândia | 20ø45'41"S 54ø50'37"W | 507 m  Cerrado | D. poecilotis (r)  G. agilis (r) | -  - | 5 transect lines (13 live traps per line) [4] |
| 29. Fazenda Sossego - Campo Grande | 20ø29'05"S 54ø29'58"W | 619 m  Cerrado | G. agilis (v)  T. macrurus (v) (ph) | 555 (M)  554 (F) | 5 transect lines (13 live traps per line) [4] |
| 30. Anhanduí - Campo Grande | 20ø59'40"S 54ø30'23"W | 420 m | D. poecilotis (r)  D. aurita (r) | -  - | 5 transect lines (12 live traps per line) [4] |
| 31. Fazenda Bela Vista - Nova Alvorada do Sul | 21ø25'15"S 54ø24'02"W | 455 m  Cerrado | D. poecilotis (v) | 245 (M) | 5 transect lines (20 live traps per line)  1 transect line (4 pitfall traps per line) [4] |
| 32. Fazenda Laranjeira - Nova Alvorada do Sul | 21ø35'16"S 53ø52'28"W | 350 m  Cerrado | D. poecilotis (r)  G. agilis (r) | -  - | 5 transect lines (13 live traps per line) [4] |
| 33. Fazenda Conquista - Santa Rita do Pardo | 21ø37'08"S 52ø11'19"W | 305 m  Atlantic F. | D. poecilotis (r) | - | 5 transect lines (20 live traps per line)  1 transect line (5 pitfall traps per line) [4] |
| 34. Distrito de Rio Verde - Três Lagoas | 20ø54'03"S 52ø11'50"W | 361 m  Cerrado | D. poecilotis (r)  G. agilis (r) | -  - | 5 transect lines (13 live traps per line) [4] |
| 35. Estância Figueira - Três Lagoas | 20ø48'50"S 51ø43'50"W | 325 m  Atlantic F. | C. agricolai (v)  G. agilis (v)  D. poecilotis (r) | 089 (M)  085 (M)  - | 5 transect lines (18 live traps per line)  1 transect line (7 pitfall traps per line) [4] |
| 36. Granja - Exército Brasileiro em Bela Vista | 22ø04'45"S 56ø33'00"W | 238 m  Cerrado | D. poecilotis (r)  G. agilis (r)  M. rapposa (r) (ph)  T. macrurus (r) | -  -  -  - | 5 transect lines (13 live traps per line) [4] |
| 37. Fazenda Redomão - Bela Vista | 22ø03'10"S 56ø25'15"W | 276 m  Cerrado | M. rapposa (r) | - | 5 transect lines (10 live traps per line) [4] |
| 38. Fazenda Lagoão - Itaporã | 22ø01'29"S 54ø47'31"W | 354 m  Atlantic F. | C. chacoensis (v) | MHNCI | 2 transect lines (5 live traps per line) [4] |
| 39. Fazenda Monjolo - Douradina | 22ø05'56"S 54ø35'20"W | 309 m  Atlantic F. | G. agilis (v)  D. poecilotis (r) | 553 (F)  - | 2 transect lines (5 live traps per line) [4] |
| 40. Fazenda Inho - Rio Brilhante | 21ø54'49"S 54ø32'40"W | 285 m  Atlantic F. | C. agricolai (v)  D. poecilotis (r) | 019 (M)  - | 2 transect lines (5 live traps per line) [4] |
| 41. Escola Agrícola - Amambai | 23ø05'06"S 55ø25'38"W | 445 m  Atlantic F. | G. agilis (v) | 469 (F) | 1 transect line (5 pitfall traps) [2] |
| 42. Fazenda Alegrete - Amambai | 23ø01'12"S 55ø04'21"W | 383 m  Atlantic F. | D. poecilotis (r)  G. agilis (r) | -  - | 5 transect lines (13 live traps per line) [4] |
| 43. Fazenda Campanário - Laguna Carapã | 22ø51'08"S 54ø59'49"W | 364 m  Atlantic F. | D. poecilotis (r) (ph) | - | 5 transect lines (13 live traps per line) [4] |
| 44. Parque Estadual das Várzeas do Rio Ivinhema – Naviraí | 22ø51'52"S 53ø38'38"W | 303 m  Atlantic F. | G. agilis (v)  M. murina (v) | 613 (M)  612 (M) | 5 transect lines (13 live traps per line) [4] |
| 45. Coxim - 47o. Batalhão do Exército | -18.522285  -54.713029 | 245 m Cerrado | G. agilis (v) | 912 (-) | 2 transect lines (12 live traps per line) [24] |
| 46. Novo Horizonte do Sul - Fazenda Japema | -22.545563  -53.889819 | 387 m  Atlantic F. | M. murina (v) | 903 (M) | 2 transect lines (15 live traps per line) [4] |
| 47. Aquidauana - Pousada das Amoras (São Lourenço) | 19ø37'44''S  55ø36'12''W | 130 m  Pantanal | G. agilis (v) | 509 (F) | 1 transect lines (30 live traps per line)  1 transect lines (6 pitfall traps per line) [4] |
| 48. Caracol – Exército de Bela Vista | -22.213720  -57.301068 | 127 m  Cerrado | P. canus (r) (ph) | - | 2 transect lines (15 live traps per line) [4] |
| 49. Águas de Miranda, Bonito, MS | 20ø39'58"S  56ø18'40"W | Cerrado | C. chacoensis (v)  M. domestica (r) | -  - | 2 transect lines (10 live traps per line) [20] |
| 50. Águas de Miranda, Bonito, MS | 20ø41'38"S  56ø18'44"W | Cerrado | G. agilis (v)  M. rapposa (v)  T. macrurus (r) | UFMT  625 (M)  - | 2 transect lines (10 live traps per line) [20] |
| 51. Fazenda Campo Alegre, Miranda, MS | 20ø30'02"S  56ø07'30"W | Cerrado | G. agilis (r)  M. domestica (r)  T. macrurus (v) | -  -  636 (M) | 2 transect lines (10 live traps per line) [20] |
| 52. Anastácio, MS | 20ø40'38"S 55ø53'18"W | Cerrado | D. leucotis (r)  G. agilis (r) | -  - | 2 transect lines (10 live traps per line) [20] |
| 53. Fazenda Estrela D’Alva, Bonito, MS | 21ø13'45"S 56ø18'15"W | Cerrado | D. poecilotis (r)  G. agilis (r)  M. domestica (r)  T. macrurus (r) | -  -  -  - | 2 transect lines (10 live traps per line) [20] |
| 54. Colônia Padroeira do Brasil, Anastácio, MS | 20ø44'18"S  55ø53'42"W | Cerrado | D. poecilotis (r)  G. agilis (v)  T. macrurus (v) | -  UFMT  UFMT | 2 transect lines (10 live traps per line) [20] |
| 55. Águas de Miranda, Bonito, MS | 20ø40'10"S 56ø19'16"W | Cerrado | G. agilis (v)  D. poecilotis (r)  M. domestica (r)  T. macrurus (r) | UFMT | 2 transect lines (10 live traps per line) [20] |
| 56. Fazenda Sucuri, Terenos, MS | 20ø36'23"S 54ø58'54"W | Cerrado | D. poecilotis (r)  G. agilis (v)  T. macrurus (v) | -  UFMT  UFMT | 2 transect lines (10 live traps per line) [20] |
| 57. Campo, Anastácio, MS | 20ø28'54"S 56ø06'21"W | Cerrado | G. agilis (r)  T. macrurus (v)  D. poecilotis (r) | UFMT  UFMT  - | 2 transect lines (10 live traps per line) [20] |
| 58. Fazenda Garuva, Terenos, MS | 20ø21'45"S 55ø06'41"W | Cerrado | D. poecilotis (r)  G. agilis (v)  M. domestica (r)  T. macrurus (r) | -  UFMT  -  - | 2 transect lines (10 live traps per line) [20] |
| 59. Coqueiro, Bonito, MS | 21ø10'47"S  56ø21'51"W | Cerrado | D. poecilotis (r)  G. agilis (r) | -  - | 2 transect lines (10 live traps per line) [20] |
| 60. Colônia Padroeira do Brasil, Anastácio, MS | 20ø45'59"S  55ø53'00"W | Cerrado | D. poecilotis (r)  G. agilis (r) | -  - | 2 transect lines (10 live traps per line) [20] |
| 61. Lalima, Bonito, MS | 20ø40'52"S 56ø21'23"W | Cerrado | D. poecilotis (r)  G. agilis (r) | -  - | 2 transect lines (10 live traps per line) [20] |
| 62. Fazenda Coqueiral II, Terenos, MS | 20ø33'11"S 54ø58'54"W | Cerrado | G. agilis (v)  D. poecilotis (r) | UFMT  - | 2 transect lines (10 live traps per line) [20] |
| 63. Fazenda Maria, Anastácio, MS | 20ø29'17"S 56ø05'15"W | Cerrado | D. poecilotis (r)  G. agilis (r)  T. macrurus (r) | -  -  - | 2 transect lines (10 live traps per line) [20] |
| 64. Fazenda Santa Helena, Terenos, MS | 20ø20'44"S 55ø13'20"W | Cerrado | G. agilis (r)  T. macrurus (r) | -  - | 2 transect lines (10 live traps per line) [20] |
| 65. Cássio, Bonito, MS | 21ø10'51"S  56ø20'55"W | Cerrado | T. macrurus (r)  G. agilis (r)  D. poecilotis (r) | -  -  - | 2 transect lines (10 live traps per line) [20] |
| 66. Fazenda Ouro Verde, Bonito, MS | 20ø40'50"S 56ø17'27"W | Cerrado | D. poecilotis (r)  G. agilis (v) | -  UFMT | 2 transect lines (10 live traps per line) [20] |
| 67. Estância Império, Terenos, MS | 20ø39'47"S 55ø00'50"W | Cerrado | C. chacoensis (v)  D. poecilotis (r)  G. agilis (r)  M. domestica (r) | UFMT  -  -  - | 2 transect lines (10 live traps per line) [20] |
| 68. Campo, Anastácio, MS | 20ø30'05"S 56ø07'17"W | Cerrado | G. agilis (r)  M. domestica (r)  T. macrurus (v) | -  -  UFMT | 2 transect lines (10 live traps per line) [20] |
| 69. Fazenda Santa Helena, Terenos, MS | 20ø20'43"S 55ø12'58"W | Cerrado | G. agilis (r)  T. macrurus (r) | -  - | 2 transect lines (10 live traps per line) [20] |
| 70. Bonito, MS | 21ø11'51"S 56ø18'35"W | Cerrado | D. poecilotis (r)  G. agilis (v)  T. macrurus (r) | -  UFMT  - | 2 transect lines (10 live traps per line) [20] |
| 71. Bonito, MS | 21ø10'16"S  56ø21'55"W | Cerrado | D. poecilotis (r)  G. agilis (v)  M. rapposa (r)  T. macrurus (r) | -  UFMT  -  - | 2 transect lines (10 live traps per line) [20] |
| 72. Miranda, MS | 20ø31'57"S 56ø10'54"W | Cerrado | D. poecilotis (r)  G. agilis (v)  P. canus (r) | -  UFMT  - | 2 transect lines (10 live traps per line) [20] |
| 73. Terenos, MS | 20ø37'13"S 55ø04'02"W | Cerrado | D. poecilotis (r)  G. agilis (r) | -  - | 2 transect lines (10 live traps per line) [20] |
